# Novel Insights on the Synergistic Mechanism of Action Between the Polycationic Peptide Colistin and Cannabidiol Against Gram-Negative Bacteria

**DOI:** 10.3390/pharmaceutics18010051

**Published:** 2025-12-30

**Authors:** Merlina Corleto, Matías Garavaglia, Melina M. B. Martínez, Melanie Weschenfeller, Santiago Urrea Montes, Martin Aran, Leonardo Pellizza, Diego Faccone, Paulo C. Maffía

**Affiliations:** 1 Laboratorio de Aplicaciones Biotecnológicas y Microbiología (LAByM), Instituto de Biotecnología, Universidad Nacional de Hurlingham, Teniente Origone 151, Villa Tesei 1425, Argentina; merlina.corleto@unahur.edu.ar (M.C.); matias.garavaglia@unahur.edu.ar (M.G.); melina.martinez@unahur.edu.ar (M.M.B.M.); melanie.weschenfeller@unahur.edu.ar (M.W.); santiago.montes@unahur.edu.ar (S.U.M.); 2 Consejo Nacional de Investigaciones Científicas y Técnicas (CONICET), Godoy Cruz 2290, Buenos Aires 1425, Argentina; dfaccone@anlis.gob.ar; 3 Laboratorio de Resonancia Magnética Nuclear, Fundación Instituto Leloir, IIBBA-CONICET, Av. Patricias Argentinas 435, Buenos Aires 1405, Argentina; maran@leloir.org.ar (M.A.); lpelliza@leloir.org.ar (L.P.); 4 Servicio Antimicrobianos, Instituto Nacional de Enfermedades Infecciosas-ANLIS “Dr. Carlos G. Malbrán”, Buenos Aires 1282, Argentina

**Keywords:** colistin, cannabidiol, synergy

## Abstract

**Background/Objectives**: Colistin (polymyxin E) has re-emerged as a last-hope treatment against MDR Gram-negative pathogens due to the development of extensively drug-resistant Gram-negative bacteria. Unfortunately, rapid global resistance towards colistin has emerged, which represents a major public health concern. In this context (CBD), a lipophilic molecule derived from Cannabis sativa, exhibits antimicrobial activity mainly against Gram-positive bacteria but is generally ineffective against Gram-negative species. However, synergistic antibacterial activity between CBD and polymyxin B has been reported. The objective of this work is to analyze the colistin–CBD synergy against clinically relevant Gram-negative isolates displaying diverse mechanisms of colistin resistance and to explore the basis of the possible mechanism of action involved in the first steps of this synergy. **Methods**: Microbiological assays, minimal inhibitory concentration, cell culture, synergy tests by checker board and time kill, biofilm inhibition evaluation by crystal violet and MTT, SEM (scanning electron microscopy), molecules interaction analysis by nuclear magnetic resonance (NMR). **Results**: The colistin–CBD combination displayed synergy in colistin resistant Gram-negative bacteria and also disrupted preformed biofilms and killed bacteria within them. Time-kill assays revealed rapid bactericidal activity and SEM showed mild surface alterations on bacterial outer membranes after sublethal colistin monotherapy. Furthermore, a series of sequential treatment assays on colistin-resistant Escherichia coli showed that simultaneous exposure to both compounds was required for activity, as introducing a washing step between treatments abolished the antibacterial effect. In order to obtain deeper insight into this mechanism, NMR analyses were performed, revealing specific molecular interactions between CBD and colistin molecules. **Conclusions**: These results provide evidence for the first time that both molecules engage through a specific and structurally meaningful interaction and only display synergy when acting together on colistin-resistant bacteria.

## 1. Introduction

Antibiotic resistance has emerged as a global health concern, challenging the effectiveness of critical medical treatments and threatening the ability to control common and serious infections [1]. Among the strategies used in clinical practice to combat resistant infections, polymyxins have been considered a last line of defense thanks to their potent antimicrobial activity. These molecules are cationic lipopeptides with a core structure of a cyclic heptapeptide and a linear tripeptide side chain. They are characterized by a high content of the non-proteinogenic amino acid α,γ-diaminobutyric acid (Dab), which gives them a positive charge at physiological pH [2]. Two closely related polymyxin antibiotics are used clinically, colistin (polymyxin E) and polymyxin B, which share a high degree of structural similarity [3,4]. As colistin is being administered routinely, the incidence of resistance is escalating, consequently increasing mortality rates among septic patients [5].

The mechanism of action of colistin, like the rest of polymyxins, is well known: polymyxin B promotes the destabilization of LPS leading to the disruption of the bacterial cell envelope. Most of the polymyxin resistance mechanisms identified in Gram-negative bacteria involve changes in the structure of lipopolysaccharide (LPS), as polymyxins initially interact with the negatively charged lipid component A of LPS [6].

Recently, mobilized colistin resistance (*mcr*) genes have been identified in many species of *Enterobacteriaceae* and *Acinetobacter*. Among them, *Escherichia coli*, *Klebsiella pneumoniae*, and *Acinetobacter baumannii* have led to the increased use of polymyxin antibiotics, which are often the only viable last-resort therapeutic option [4,7,8]. This critical situation highlights the importance of identifying new therapeutic alternatives.

Within this context, the search for natural sources of antimicrobial agents has gained relevance, and phytotherapeutics have emerged as a promising field of research. Among the most notable is cannabis, a plant that has been widely studied for its chemical compounds, known as cannabinoids, which include cannabidiol (CBD) and delta-9-tetrahydrocannabinol (THC), and the different terpenes and flavonoids, among others [9,10].

Interest in cannabinoids as a potential antimicrobial agent has increased greatly due to growing evidence of its medicinal properties, as well as its ability to influence the endocannabinoid system of the human body. Among the main active ingredients is CBD, a lipophilic and non-psychoactive molecule that has demonstrated notable antimicrobial properties. Its lipophilicity confers this molecule an affinity for cell membranes, which may influence its ability to penetrate and affect the structure of bacteria, principally Gram-positive bacteria [11,12,13].

On the other hand, its effectiveness against Gram-negative bacteria has been less evident, which raises the need to explore strategies that enhance its activity [14].

Regarding its mode of action, it is supposed to interact mainly with bacterial membranes, as determined in biophysical studies with model membranes. In these studies, CBD could modify their transition temperature, enthalpy of cohesion, and cooperativity, which indicates a strong alteration of the membranes [15], although other non-membranolytic activity has been proposed [16].

In this context, synergy emerges as a promising alternative to improve the efficacy of current antimicrobial treatments and also the possibility to diminish the dose of the antibiotic. This is particularly important for those antibiotics that display some toxicity at high concentration, like colistin [17].

In this study, we evaluate the synergy between colistin (polymyxin E) and CBD in colistin-resistant strains of Gram-negative bacteria, originating from clinical isolates with different types of resistance to polymyxins (*mcr-1* encoded, chromosomic encoded, or intrinsic resistance). Bacterial biofilms are also quantified and *E. coli* outer membrane is analyzed by scanning electron microscopy (SEM). Furthermore, the potency and efficacy of the synergy are determined by time-kill assay on two *E. coli* strains harboring different colistin-resistant mechanisms.

In an attempt to unveil the possible mode of action of the colistin–CBD combination, microbiological assays are performed in which CBD and colistin are alternatively incubated with the bacteria, with or without intermediate washing steps. In these experiments, we observe that the two compounds have to be together at the same time when incubated with bacteria for the antimicrobial action to take place, perhaps contrary to the idea of a sequential mode of action. To address this issue, we evaluate the possible interaction between these two antimicrobials using NMR (nuclear magnetic resonance) and we observe that CBD engages colistin through a specific and structurally meaningful interaction in a stoichiometric relationship of 1:1.

This work provides evidence for the first time that colistin and CBD strongly interact between each other and only display synergistic antimicrobial activity when acting together at the same time, possibly in a colistin–CBD single complex or a cooperative membrane insertion mode of action against colistin-resistant Gram-negative bacteria.

## 2. Materials and Methods

### 2.1. Strains and Growth Conditions

Bacteria used in this work were clinical isolates identified and characterized at the ANLIS “Carlos G. Malbrán” Institute. Strains used in this work were colistin-resistant *mcr-1* positive: *E. coli* M15049 and M15224, *Salmonella typhimurium* M22399, *K. pneumoniae* M21664, or colistin-resistant *mcr-1* negative: *E. coli* M27666, *K. pneumoniae* M28644, *Salmonella* spp. M28629, and *Acinetobacter baumannii* M27167. The strains were grown in Mueller–Hinton broth at 37 °C, 200 rpm, for 24 h.

### 2.2. Cannabidiol and Antibiotics Used

The cannabidiol used in this work was a kind gift from the Kilab S.A. company (Buenos Aires, Argentina). The drug was 98.9% pure lyophilized CBD, and DMSO pure (dimethylsulphoxide) was used to dissolve it.

Colistin sulfate, gentamicin, and meropenem (Supelco^®^, Sigma Aldrich, St. Louis, MO, USA) were used as antibiotics for control and synergy tests.

### 2.3. Cytotoxicity Assay by MTT

#### 2.3.1. Cell Culture

The human alveolar basal epithelial adenocarcinoma cell line A549 (ATCC CCL-185) was cultured in complete RPMI medium supplemented with 10% fetal bovine serum (FBS; Internegocios S.A., Mercedes, Argentina). Cytotoxic effects were indirectly assessed using the colorimetric MTT assay (3-[4,5-dimethylthiazol-2-yl]-2,5-diphenyltetrazolium bromide). The A549 cell line was kindly provided by the Instituto de Virología e Innovaciones Tecnológicas (IVIT; INTA–CONICET, Hurlingham, Buenos Aires, Argentina). For the assay, cells were seeded into 96-well flat-bottomed plates at a density of 1 × 10^4^ cells per well and incubated for 48 h at 37 °C in a humidified atmosphere containing 5% CO_2_. Cells were subsequently treated with colistin in combination with CBD, while colistin alone and CBD alone were included as control treatments.

#### 2.3.2. MTT Assay

Cell viability was assessed using the MTT reduction assay. After the treatment period, culture supernatants were removed and cells were washed with phosphate-buffered saline (PBS). Subsequently, 100 µL of MTT solution (0.5 mg/mL in PBS) was added to each well, and plates were incubated at 37 °C in a humidified atmosphere containing 5% CO_2_ for 0.5–4 h. During this incubation, metabolically active cells reduced MTT to insoluble formazan crystals, which were confirmed by light-field microscopy (Zeiss Primovent, Oberkochen, Germany). At the end of the incubation, the MTT solution was aspirated and 200 µL of dimethyl sulfoxide (DMSO) was added to each well to solubilize the formazan crystals. Plates were gently shaken to ensure complete dissolution, and absorbance was measured at 570 nm using a microplate reader (RT2100, Rayto Life and Analytical Sciences Co., Ltd., Shenzhen, China). Each treatment was performed in triplicate. Cell viability was expressed as a percentage relative to untreated control cells, which were defined as 100% viability. Data visualization was carried out using GraphPad Prism version 5.0.

### 2.4. Minimum Inhibitory Concentration (MIC)

The MIC was determined using standard microdilution assays, adhering to the Clinical and Laboratory Standards Institute (CLSI) recommendations [18,19]. Mueller–Hinton Broth (MHB, Britania, Buenos Aires, Argentina) was utilized for all strains. In brief, 96-well U-bottomed plates were employed, with drug treatments added and serially diluted, ranging from 32 to 0.5 µg/mL (except for *A. baumannii*, for which colistin concentration reached up to 128 µg/mL). An inoculum of 5 × 10^5^ CFU/mL was introduced, and the plates were incubated at 37 °C for 24 h. The results were assessed visually, and optical density (OD) measurements were taken to quantify and graph the obtained data.

### 2.5. Synergy Assay

To evaluate the possible synergistic activity of CBD with colistin, a screening by broth microdilution method following the checkerboard method for synergy was performed.

The checkerboard assay was performed following [20] with some modifications. Briefly, in flat-bottomed 96-well plates, two fixed concentrations of colistin (0.5× MIC and 0.25× MIC for each strain) were established, along with decreasing concentrations of CBD (32–0.5 µg/mL). After ON incubation at 37 °C, optical density measurements at 600 nm were recorded. The interaction between cannabidiol (CBD) and different antimicrobials was evaluated in flat-bottomed 96-well plates. CBD concentrations ranged from 0.5 to 32 µg/mL against each antibiotic. The antimicrobial gentamicin ranged from 0.125 to 8 µg/mL and meropenem from 0.002 to 0.125 µg/mL.

The term “minimal effective antibiotic concentration” (MEAC) was used in these synergy assays, corresponding to the concentration of the antibiotic colistin in the combination with CBD that inhibited bacterial growth.

### 2.6. Time-Kill Assay

Synergistic activity was assessed using the time-kill assay. Briefly, exponential phase cultures grown at 37 °C were adjusted by optical density and diluted in Mueller–Hinton broth to a final inoculum of 5 × 10^5^ CFU/mL. Assays were performed in 96-well microplates with a final volume of 200 µL per well. Bacterial suspensions were exposed to the corresponding antimicrobial conditions: growth control, cannabidiol (CBD) alone, colistin sub-MIC (Col) alone, the CBD–Col combination, and colistin at its MIC. For *E. coli* M27666, the conditions tested were CBD 8 µg/mL, Col 3 µg/mL, the combination of both, and Col MIC; for *E. coli* M15224, CBD 4 µg/mL, Col 2.5 µg/mL, the combination of both, and Col MIC.

Aliquots were collected at 0, 1, 2, 3, 4, 8, and 24 h. Viable counts were determined by tenfold serial dilutions followed by spot plating on LB agar (Miles and Misra method). Plates were incubated at 37 °C for 24 h before colony enumeration. Synergy is defined as a ≥2 log_10_ reduction in CFU/mL for the combination compared to the most active single agent, at 24 h.

### 2.7. Scanning Electron Microscopy (SEM)

Sample preparation for scanning electron microscopy (SEM) was carried out following the protocol described in [18], with minor modifications. *Escherichia coli* M15224 cells were cultured in Mueller–Hinton (MH) broth to the exponential growth phase. Cultures were centrifuged at 10,000× *g* for 1 min, after which the pellets were collected, washed twice with phosphate-buffered saline (PBS), and resuspended to an optical density at 600 nm (OD_600_) of 0.2. Bacterial suspensions were incubated at 37 °C for 1 h in the presence of colistin at 0.25× MIC, the colistin–CBD combination (synergy condition), CBD alone, or untreated growth controls. Following incubation, cells were centrifuged at 7000× *g* for 10 min, and the resulting pellets were recovered and washed twice with PBS. Fixation was performed using 2.5% glutaraldehyde at 4 °C overnight, followed by two additional PBS washes. Samples were subsequently dehydrated through a graded ethanol series (50%, 70%, 90%, and 100%; 10 min each) and transferred to 100% acetone. Finally, specimens were gold-coated and analyzed using a Carl Zeiss NTS SUPRA 40 scanning electron microscope.

### 2.8. Sequential Incubation of Colistin and Cannabidiol on E. coli M15224

In this antimicrobial assay, the compounds were added sequentially with a defined time interval, in flat-bottomed 96-well plates, with a final volume of 200 µL per well. In the first condition, CBD was added at concentrations ranging from 0.5 to 32 µg/mL, and after 30 min of incubation at 37 °C, colistin was added at subinhibitory concentrations (1/4 and 1/2 of the previously determined MIC). In parallel, the inverse condition was tested, adding colistin first and then CBD following the same experimental scheme. Controls included each antimicrobial alone, the MIC of colistin, and the simultaneous addition of both antimicrobials, as well as growth controls (without antimicrobials) and sterility controls (without inoculum). Plates were incubated overnight at 37 °C, and antimicrobial activity was evaluated by measuring optical density (OD) at 600 nm. The assay was performed in triplicate with four replicates per condition.

### 2.9. Sequential Incubation of Colistin and Cannabidiol with Intermediate Washing Step on E. coli M15224

To determine whether the synergistic effect required the simultaneous presence of both antimicrobials or if the initial exposure caused a persistent alteration in the bacterial membrane, a second protocol including an intermediate washing step was carried out.

In this case, bacteria were incubated in 15 mL conical tubes with a final volume of 1 mL, treated first with CBD or colistin for 30 min at 37 °C. Subsequently, two washes were performed with sterile saline solution, and the pellet was resuspended with the second treatment. Afterwards, 200 µL of each condition were transferred to flat-bottomed 96-well plates and incubated overnight at 37 °C. Controls included individual treatments, the simultaneous combination of both antimicrobials, the MIC of colistin, and the growth control without antimicrobials, all processed following the same washing steps. The concentrations used in this second assay corresponded to the lowest CBD and colistin concentrations at which synergy had previously been observed. After incubation, antimicrobial activity was assessed by measuring OD at 600 nm.

### 2.10. NMR Studies

In order to evaluate the interaction between the CBD and colistin molecules, NMR experiments were performed at 298 K in a Bruker 600 MHz Avance III spectrometer operating at a proton frequency of 600.1 MHz (Bruker Instruments, Inc., Bellerica, MA, USA). Then, 1H-NMR spectra with multiple solvent suppression were acquired using a standard Bruker 1D NOESY pulse program with presaturation of solvent signals during the relaxation delay, mixing time, and spoil gradients (noesygpps1d). Data were collected using the following experimental parameters: 64 scans, 1.8 s relaxation delay, 1.36 s acquisition time, 20 ppm spectral width, 10 ms mixing time, and 32 K acquisition points. Spectra were Fourier-transformed, phase- and baseline-corrected using NMRPipe [21], and referenced to TSP (1H δ = 0 ppm). For the 1H-NMR interaction study, colistin was initially prepared at a concentration of 40 μM in 60% ethanol: H2O containing 5% of deuterium dioxide (D2O). CBD, dissolved in ethanol, was then incrementally added to the colistin solution to achieve final concentrations of 4, 8, 14, 20, 45, 130, and 400 μM, respectively. Changes in chemical shifts and peak intensities were monitored to identify potential molecular interactions and conformation alterations upon colistin–CBD binding.

### 2.11. Biofilm

The activity against preformed biofilms was evaluated in flat-bottomed 96-well polystyrene microplates. Two plates were prepared by inoculating 100 µL per well of a bacterial suspension adjusted to 5 × 10^5^ CFU/mL in Mueller–Hinton broth (Laboratorios Britania S.A, Buenos Aires, Argentina), followed by incubation at 37 °C for 24 h. Afterwards, the supernatant was removed, and 100 µL of MH broth containing the different treatments were added. After a further 24 h of incubation, optical density was measured at 595 nm to quantify bacterial growth. One plate was then used to evaluate total biofilm biomass, and the second was used to determine the number of viable bacteria within the biofilm.

### 2.12. Crystal Violet

Biofilm biomass was quantified using the crystal violet staining method. Following incubation, culture supernatants were carefully aspirated, and the resulting biofilms were gently washed twice with 100 µL of saline solution to remove non-adherent (planktonic) cells. The attached biofilms were then fixed with 100 µL of absolute methanol for 15 min, after which staining was performed using 100 µL of 1% (*v*/*v*) crystal violet for 5 min. Excess stain was discarded, and wells were rinsed twice with 200 µL of distilled water. Plates were subsequently air-dried at 37 °C for 30 min. To solubilize the bound dye, 100 µL of 33% (*v*/*v*) acetic acid was added to each well, samples were gently mixed, and absorbance was measured at 595 nm using a microplate reader (RT-6000, Rayto Life and Analytical Sciences Co., Ltd.).

### 2.13. MTT Assay for Bacteria

Bacterial viability within biofilms was evaluated using the MTT assay (3-(4,5-dimethylthiazol-2-yl)-2,5-diphenyltetrazolium bromide; Thermo Fisher Scientific Inc., Massachusetts, Waltham, MA, USA), which is based on the reduction of MTT to purple formazan crystals by metabolically active cells. After incubation, culture supernatants were carefully aspirated and the biofilms were washed three times with saline solution. Subsequently, 100 µL of 0.05% (*w*/*v*) MTT solution was added to each well, and plates were incubated at 37 °C for 3 h in the dark. Following incubation, the MTT solution was removed and the resulting formazan crystals were solubilized with dimethyl sulfoxide (DMSO). Absorbance was recorded at 595 nm using a microplate reader (RT-6000, Rayto Life and Analytical Sciences Co., Ltd.).

## 3. Results

### 3.1. Cytotoxicity Assay

**Figure 1:**
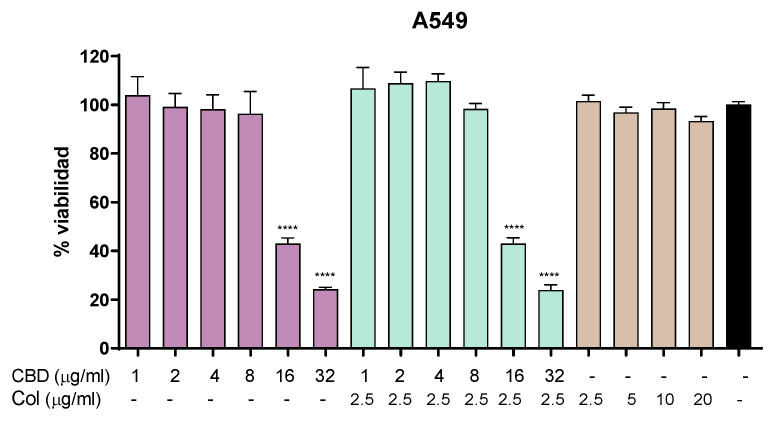
Cytotoxicity in A549 cell line. MTT assay showing cell viability after 24 h of treatment with CBD, colistin, or the combination. One-way ANOVA followed by Dunnett’s post hoc test, with each condition compared to the control of untreated cells (black bar); *n* = 5. Statistical significance: **** *p* < 0.0001.

In order to evaluate the possible cytotoxic effect of CBD and colistin in vitro, we performed an MTT assay in A549 cell line. In these experiments, we observed that CBD displayed no cytotoxic effect in the range from 1 to 8 µg/mL, and it began to show toxic effects to this cell line at 16 µg/mL. On the other hand, colistin alone did not display any toxic effect at the concentrations tested (2.5–20 µg/mL). When tested in combination, CBD and colistin showed toxicity at the same concentrations of CBD alone (Figure 1).

### 3.2. Synergistic Activity

Pure lyophilized CBD was evaluated in a wide range of concentration against a group of GN bacteria. In those experiments we found no antimicrobial activity of CBD alone, in good agreement with previous reports that stated that this molecule displayed activity only in GP bacteria [13].

A checkerboard-like assay was performed to determine synergy on different strains of colistin-resistant *E. coli*, *K. pneumoniae*, *Salmonella* spp., and *A. baumannii.* As seen in Table 1, one *Salmonella*, one *K. pneumoniae*, and two *E. coli* strains displayed colistin resistance mediated by the *mcr-1* gene located in a plasmid, while another group of the same bacteria and *A. baumannii* strain showed resistance through chromosomal mutations.

In a first assay, the MIC of colistin alone was determined for each strain and then in combination with CBD, starting at 32 µg/mL.

Under these conditions, it was seen that in some cases the MEAC of colistin dropped to ½, in others ¼, and in others to 1/8 of the MIC of colistin alone. Based on these results, and given that the toxicity of CBD becomes evident above 16 µg/mL, it was decided to evaluate the lower MEAC of colistin at a fixed concentration with different concentrations of CBD, with the aim of reducing the concentration of this last compound in the drug combination to the minimum possible.

**Figure 2:**
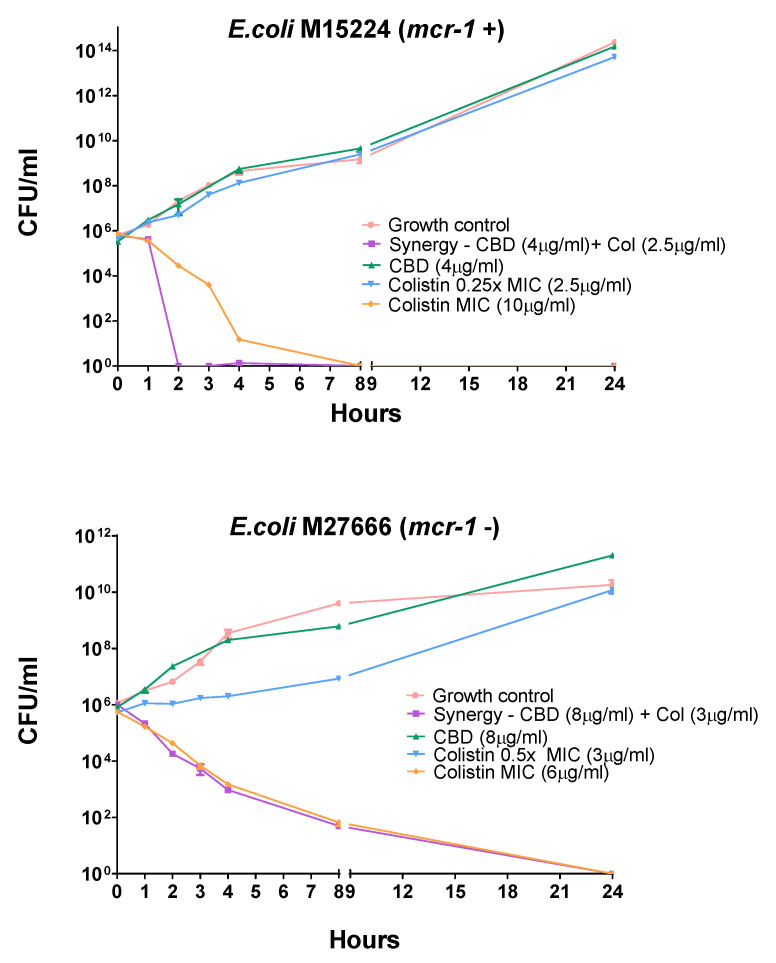
Time-kill assay for two *E. coli* strains harboring different colistin-resistant mechanisms. Each agent was tested alone at concentrations below the MIC (2.5 µg/mL for colistin). CBD did not display any antimicrobial activity in a large range of concentrations; for that reason, there is no CBD curve at its MIC. The time-kill curve of colistin at its MIC (6 or 10 µg/mL) is also depicted.

In these experiments, we observed that colistin and CBD displayed synergy against different GN species between 0.5 and 4 µg/mL of CBD at a fixed concentration of colistin (Table 1 and Figure 2). The synergy seems to be independent of the different mechanism of resistance in each bacterial species, in light of the lack of correspondence between *mcr-1*+ and *mcr-1*− and the sensitivity to the combination of CBD–colistin.

### 3.3. Evaluation of Synergy by Time-Kill Assay

The time-kill assay can be extremely useful to study the dynamics of synergism for a combination of agents by determining the number of viable bacteria remaining over time after exposure to each individual agent and the combination of them. Synergism in the time-kill assay is defined as a ≥2-log_10_ CFU/mL decrease by the combination compared to the most active single agent [22].

A time-kill assay on two colistin-resistant *E. coli* strains M15224, *mcr-1* positive, and *E. coli* M27666, *mcr-1* negative, was carried out.

In these experiments, as seen in Figure 2, the combination of compounds displayed faster activity than colistin alone in *E. coli* 15224 (*mcr-1*+); however, *E. coli* 27666 (*mcr-1*−) showed a similar effect. It is also noteworthy that each single agent, at the concentration used in combination, did not show significant activity at 24 h compared to the growth control.

### 3.4. Disruption of Preformed Biofilms

**Figure 3:**
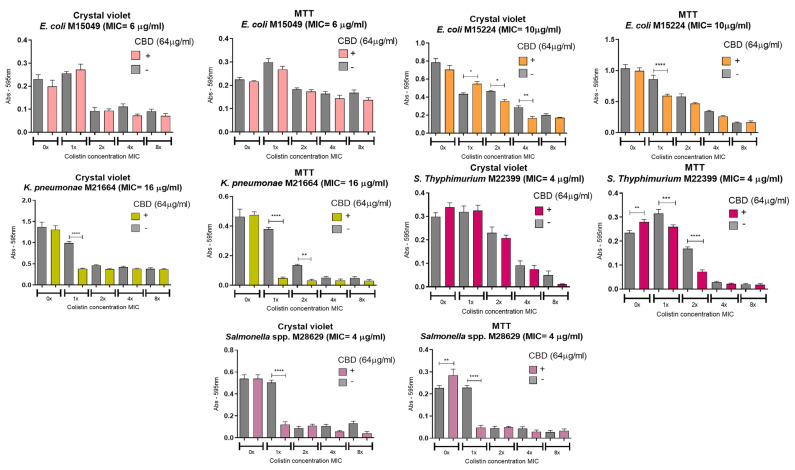
Anti-biofilm activity of CBD and colistin. Each strain was cultured for 48 h to develop biofilm and then treated with the compounds, alone or in combination, at a fixed concentration of CBD and variable concentrations of colistin (expressed as multiples of the MIC for colistin alone). The grey bar in each graph shows the bacterial growth with the indicated concentration of colistin alone with no CBD. One-way ANOVA followed by Bonferroni’s post hoc test, comparing CBD− and CBD+ conditions for each concentration of colistin; *n* = 8. Statistical significance: * *p* < 0.05, ** *p* < 0.01, *** *p* < 0.001, **** *p* < 0.0001.

The effect of cannabidiol (CBD), colistin, and their combination on preformed bacterial biofilms was assessed using crystal violet (CV) and MTT assays. After 48 h of biofilm formation, the treatments were added and incubated for an additional 24 h. CBD was tested at 64 µg/mL, while colistin was used at concentrations ranging from the MIC to 8 × MIC (Figure 3). Both agents, and particularly colistin at concentrations equal to or above the MIC, disrupted the biofilm structure and reduced bacterial cell viability. However, in several cases, the combination of CBD and colistin led to a significantly greater antibiofilm effect compared with either treatment alone. In the CV assay, a marked reduction in biofilm biomass was observed for *E. coli* M15224 at 2 × MIC and 4 × MIC of colistin, *K. pneumoniae* M21664 at 1 × MIC, and *Salmonella* spp. M28629 at 1 × MIC when CBD was combined with colistin. Similarly, MTT assays showed a significant decrease in viable bacteria within the biofilm for *E. coli* M15224 (1 × MIC), *S. typhimurium* M22399 (1 × MIC and 2 × MIC), and *Salmonella* spp. M28629 (1 × MIC). In *K. pneumoniae* M21664, the combination also reduced bacterial viability, with the most pronounced effect at 1 × MIC. This strain displayed the highest sensitivity to the synergistic effect of both compounds. Only five strains are shown in Figure 3 as *E. coli* M27666 and *K. pneumoniae* M28644 did not form detectable biofilms under these conditions. For *A. baumannii* M27167, colistin alone was sufficient to disrupt the biofilm and eradicate bacterial cells.

### 3.5. Morphological Analysis by Scanning Electron Microscopy (SEM)

**Figure 4:**
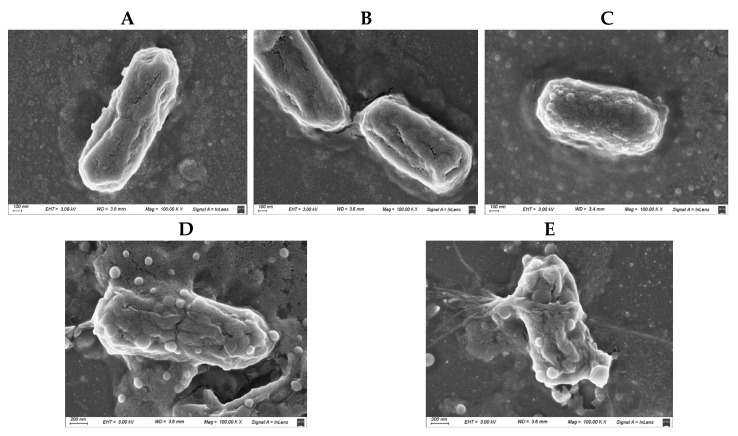
Scanning electron microscopy (SEM) images of *Escherichia coli* M15224 under different treatment conditions. (**A**) Untreated control; (**B**) cannabidiol (CBD) at the concentration used in synergy assays; (**C**) colistin at 0.25 × MIC; (**D**,**E**) CBD–colistin combination (synergy).

SEM images were used to assess morphological alterations in *Escherichia coli* M15224 under different treatment conditions (Figure 4). Untreated cells displayed the typical rod-shaped morphology, with smooth and intact surfaces showing no visible membrane damage (Figure 4A). Cells treated with cannabidiol (CBD) exhibited a similar appearance to the control, with no evident structural alterations or membrane disruption (Figure 4B). In contrast, cells exposed to subinhibitory concentrations of colistin showed surface alterations characterized by the presence of protrusions on the outer membrane (Figure 4C). Finally, the combined treatment (CBD + colistin) resulted in irreversible damage and membrane disruption (Figure 4D), supporting the synergistic effect between both compounds.

### 3.6. Evaluation of the Interaction Between Cannabidiol (CBD) and Different Antimicrobials by Checkerboard Assay

The interaction between cannabidiol (CBD) and different antimicrobials was evaluated using the checkerboard assay. Two antibiotics with distinct mechanisms of action were included: meropenem, which inhibits bacterial cell wall synthesis, and gentamicin, an aminoglycoside that interferes with protein synthesis.

**Figure 5:**
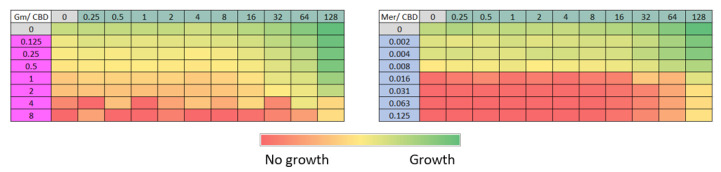
Evaluation of the interaction between cannabidiol (CBD) and different antimicrobials using the checkerboard method**.** CBD concentration ranges between 0.5 and 128 µg/mL were tested against meropenem (Mer, 0.002–0.125 µg/mL) and gentamicin (Gm, 0.125–8 µg/mL). The color gradient indicates optical density at 600 nm, from red (no growth) to green (maximum bacterial growth).

In the assays with meropenem and gentamicin (Figure 5, tables Mer/CBD and Gm/CBD), no inhibition patterns consistent with a synergistic effect between these compounds and CBD were observed. Bacterial growth occurred in almost all combinations tested, and inhibition was detected only at concentrations where the antibiotic alone was already effective.

### 3.7. Impact of the Order of Exposure on the Synergistic Interaction Between CBD and Colistin

**Figure 6:**
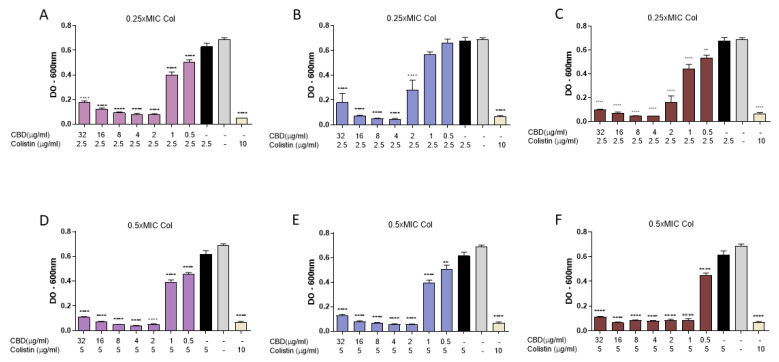
Evaluation of the impact of exposure order of cannabidiol (CBD) and colistin (Col) on *E. coli* M15224. Assays were performed in 96-well plates, measuring optical density at 600 nm after overnight incubation at 37 °C. CBD concentrations ranged from 0.5 to 32 µg/mL and colistin was tested at 0.25 × MIC or 0.5 × MIC. (**A**,**D**) CBD addition followed by Col after 30 min incubation. (**B**,**E**) Col addition followed by CBD after 30 min incubation. (**C**,**F**) Simultaneous addition of both compounds. Controls: growth without antimicrobials, each antimicrobial alone, and colistin at MIC (10 µg/mL). Data are expressed as mean ± SD of four replicates per condition. One-way ANOVA followed by Dunnett’s post hoc test, with each column compared to the control group (colistin alone at the concentration used in the synergy test, black column); *n* = 4. Statistical significance: ** *p* < 0.01; **** *p* < 0.0001.

To assess the impact of the order of exposure of colistin and CBD on *E. coli* M15224, microbiological assays were performed, varying the sequence in which the antimicrobials were added. Subinhibitory concentrations of colistin (0.25 × MIC and 0.5 × MIC) and a CBD range of 0.5–32 µg/mL were used. In the first assay, bacteria were incubated overnight with both compounds in solution, regardless of whether CBD or colistin was added first (Figure 6A,B,D,E). The control condition, in which both antimicrobials were added simultaneously, showed inhibition levels comparable to those obtained in the other tested conditions (Figure 6C,F), with no significant differences in OD_600_ among the different addition sequences.

**Figure 7:**
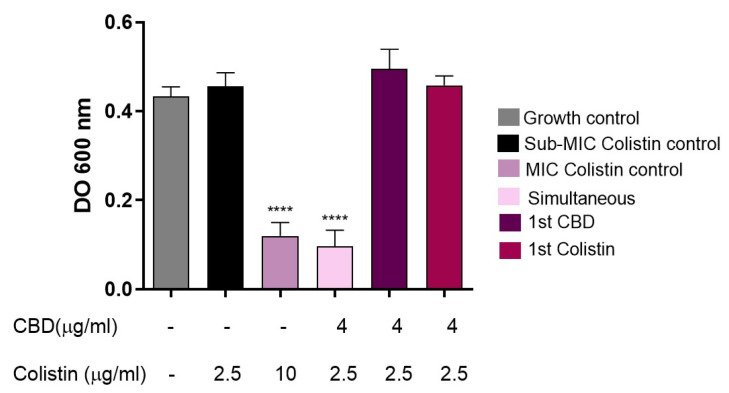
Evaluation of the effect of intermediate washing on the synergy between CBD and colistin against *E. coli* M15224. After initial incubation with the first antimicrobial (CBD or colistin) for 30 min at 37 °C, two washes with sterile saline solution were performed before the addition of the second compound. Controls: growth without antimicrobials, individual treatments, simultaneous combination, and colistin at MIC (10 µg/mL). One-way ANOVA followed by Dunnett’s post hoc test, with each column compared to the control group (colistin alone at the concentration used in the synergy test, black column); *n* = 4. Statistical significance: **** *p* < 0.0001.

Subsequently, to avoid the possible interaction of colistin and CBD molecules in supernatant solution, an intermediate washing step was implemented. In this protocol, bacteria were first incubated for 30 min with either CBD or colistin, washed twice with sterile saline solution, and then exposed to the second compound. When bacteria were subjected to this scheme (Figure 7), no inhibition of bacterial growth was observed, in contrast to the control without washing (Figure 6A) or to the condition where both antimicrobials were added simultaneously after washing bacteria, regardless of the order of addition. Under the conditions with the intermediate washing step, the combined antimicrobial effect was clearly lost.

### 3.8. Colistin–CBD Interaction Studies by NMR

To elucidate potential molecular interactions between the lipophilic cannabinoid CBD and the polycationic peptide colistin which contains both hydrophilic and lipophilic moieties, ^1^H-NMR titration experiments were performed. In these assays, increasing concentrations of CBD were gradually incorporated to a solution containing a fixed concentration of colistin, as described in Materials and Methods. Upon the addition of CBD, a new signal emerged in the amide proton region, with its intensity progressively increased with CBD concentration. The integration of this new peak as a function of CBD concentration yielded a dissociation constant (K_d_) of 36 µM. Concurrently, minor chemical shift perturbations were detected in several existing resonances, whereas changes in the aliphatic region could not be assessed due to signal overlap with solvent and CBD peaks.

**Figure 8:**
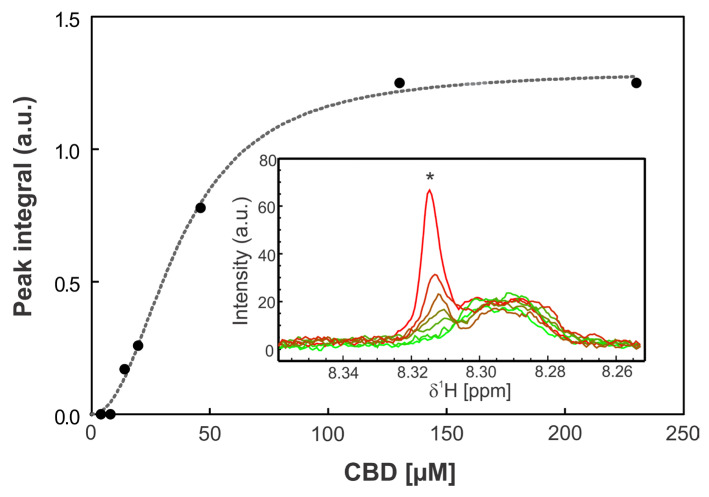
Titration assay. The graph shows the integral of the peak at 8.31 ppm as a function of CBD concentration. The inset displays the amide region of the 1H-NMR spectra at increasing CBD concentrations, where the asterisk (*) indicates the monitored peak. The spectra are shown in different colors corresponding to different CBD concentrations, with light green representing 0 µM CBD and red 230 µM CBD. Data points were fitted using a one-site binding model with a Hill slope (dashed line).

Collectively, these results provide evidence of a specific interaction between CBD and colistin, likely involving at least one residue (marked with * in Figure 8). The emergence of a new, well-defined amide proton peak upon titration of CBD into the colistin solution is a robust indicator of a binding-induced change in chemical environment. This resonance: (i) was not present in CBD-free samples, ruling out pre-existing conformers or impurities; (ii) displayed monotonic intensity growth as a function of CBD concentration, consistent with a two-state exchange regime in slow/intermediate exchange on the proton chemical shift timescale; and (iii) produced an excellent hyperbolic fit when the integrated intensity was plotted against CBD concentration, yielding a dissociation constant (K_d) of 36 µM.

## 4. Discussion

The antimicrobial effect of cannabidiol has recently gained great attention; with previous reports showing its activity against Gram-positive bacteria [11,14] and mycobacteria [23], and also displaying antibiofilm activity [14]. For Gram-negative bacteria, CBD showed no activity but displayed an interesting synergy with polymyxins [13,16]; however, the precise mode of action of this combination is still unclear. In this work, we evaluated, in a group of clinically relevant colistin-resistant Gram-negative strains, CBD, colistin, and the combination of both.

First, we analyzed the toxic effect of CBD alone in the human lung cell line A549 (Figure 1). In this cell line, CBD displayed toxicity at 16 µg/mL, in good agreement with previous reports in co-cultures of A549 with alveolar macrophages NR8383 cell line [24]. However, other authors reported hemolytic effect of CBD at 200 µg/mL in human red blood cells [14].

When tested in combination, colistin and CBD displayed synergistic effect against *E. coli*, *S. enterica*, *K. pneumoniae,* and *A. baumannii* with both chromosomal and plasmid-mediated colistin resistance (Table 1). However, we could not find any correlation between the mechanism of resistance and the concentration of CBD or colistin needed to display antimicrobial activity. The time-kill assay confirmed the synergistic activity of these two compounds, regardless of the resistance mechanism (*mcr-1*+ or *mcr-1−*), showing more than a 2 log decrease in bacterial growth for the combination in a short period of time (Figure 2). These results demonstrate the strong synergy between these two compounds with a rapid (2 h) bactericidal activity when combined, in good agreement with previous reports [13].

It is noteworthy that polymyxin-based combinations are highly variable, indicating that synergy is not uniformly expressed across all Gram-negative strains. This variability is largely attributable to differences in outer membrane composition, lipid A modifications, and adaptive resistance mechanisms. Lipid A remodeling—such as the addition of phosphoethanolamine or 4-amino-4-deoxy-L-arabinose—reduces colistin binding affinity and outer membrane disruption, thereby limiting downstream permeabilization effects [4,25]. Moreover, Gram-negative species differ substantially in LPS acylation patterns and charge density, phospholipid composition of the inner membrane, and/or ability to remodel membrane fluidity under stress. These factors directly influence the extent to which hydrophobic molecules such as CBD can partition into bacterial membranes once the OM barrier is compromised. Similar strain-dependent effects have been observed for other membrane-active combinations, including polymyxin–rifampicin and polymyxin–chloramphenicol pairs [26,27]. The magnitude and detectability of synergy are expected to be strain-specific, governed by LPS structure, resistance determinants, and membrane adaptive capacity.

Biofilm formation in bacteria plays a critical role in antimicrobial resistance, thereby complicating infection treatment and increasing the likelihood of disease relapse. In the eradication of preformed biofilm experiments, the concentration of CBD was fixed at 64 µg/mL and colistin was evaluated in a range of concentrations (as a multiple of the MIC for colistin alone). Our results vary widely between the strains, highlighting the diversity of the biofilm matrix between bacterial species and even between strains (Figure 3). Biofilms are not uniform structures, even within the same species. The relative abundance of extracellular polymeric substances (EPSs)—including polysaccharides, extracellular DNA (eDNA), proteins, and lipids—varies markedly between strains. Because the CBD–colistin interaction is fundamentally membrane- and matrix-driven rather than target-specific, relatively small strain-level differences can translate into large phenotypic effects.

Scanning electron microscopy was also performed in order to obtain images of the bacterial membrane after incubation with CBD, colistin, or the combination (Figure 4). In these images we observed that CBD alone did not affect the membrane at all, but colistin at sub-MIC concentration produced a slight disturbance (blisters or bubbles) on the surface of the bacteria external membrane. When both compounds were included together, the membrane lysis was clearly observed. The bubbles or blisters that appear in the SEM micrographs reflect the destabilization of the outer membrane induced by colistin. This phenomenon is also seen for other cationic antimicrobial peptides [28]. This membrane perturbation induces a disruption or alteration of the normal, ordered structure and packing of lipids within a cell membrane. In the case of sub-MIC concentration of colistin alone, this perturbation, although visible by SEM, is not enough to produce bacterial death.

The checkerboard method was also used to test if CBD would display synergy with other antibiotics, like meropenem or gentamicin, but no synergistic effect was observed with these two antimicrobials (Figure 5). It is noteworthy that these two antibiotics display completely different mechanisms of action than colistin; meropenem inhibits bacterial cell wall synthesis and gentamicin interferes with protein synthesis.

In several systems, reciprocal effects have been observed, where membrane insertion of the partner drug prolongs polymyxin association with the membrane, reinforcing damage. Polymyxins + hydrophobic antibiotics is perhaps a well-characterized example of cooperative membrane insertion in Gram-negative bacteria. Polymyxins (including colistin) bind to lipid A in lipopolysaccharide (LPS), displacing divalent cations and disrupting outer membrane organization [27]. This permeabilization allows otherwise-excluded hydrophobic antibiotics to insert into and traverse the outer membrane, markedly enhancing their intracellular access and activity.

In a previous study, Abichabki et al. investigated the antibacterial activity of a CBD–polymyxin B combination against Gram-negative bacteria, including polymyxin B-resistant Gram-negative bacilli. The authors proposed that the lack of intrinsic activity of CBD against Gram-negative organisms is primarily due to limited permeability across the outer membrane of the bacterial cell envelope [23]. Given the hydrophobic chemical structure of CBD, a strong interaction with membrane lipids is expected, and the cytoplasmic membrane has been identified as a potential target for cannabinoids. Based on the use of sublethal (subinhibitory) concentrations of polymyxin B in the combination, the authors suggested that CBD represents the main antibacterial component. Accordingly, a two-step mechanism of action was proposed, whereby polymyxin B initially disrupts the outer membrane of polymyxin-resistant Gram-negative bacteria, allowing subsequent penetration of CBD into the periplasmic space and interaction with the cytoplasmic membrane, ultimately leading to antibacterial activity [23].

In order to test this proposed mechanism, we incubated a colistin-resistant *E coli* sequentially with the two agents, first with colistin at sub-lethal concentration (the concentration that showed antimicrobial activity when combined with CBD) and then with CBD (Figure 6). In these experiments, with no washing step in between treatments, synergistic antimicrobial activity was observed, regardless of the order in which colistin or CBD were added.

If colistin had made a destabilization of the membrane, then CBD would enter the outer membrane and display its antimicrobial activity. However, when the experiment was repeated, but with a washing step after colistin treatment and before CBD incubation, surprisingly, no antimicrobial activity was seen (Figure 7). It is noteworthy that without washing, colistin molecules would probably be available in the supernatant to interact with CBD. This result prompted us to consider that the two molecules had to be together at the same time in order to display antimicrobial activity; perhaps acting as a whole, at least at this first step of the bacterial killing process, probably interacting with the bacterial outer membrane.

**Figure 9:**
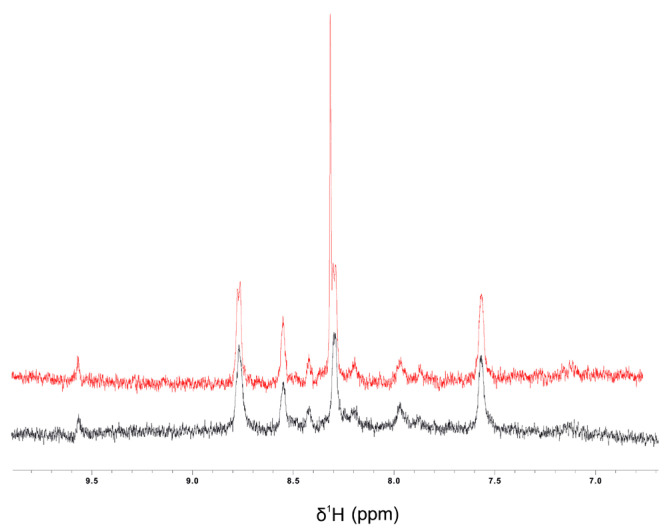
^1^H-NMR spectra of the amide region (6.5–10.0 ppm) of colistin (40 µM) in 60% ethanol: H_2_O in the absence (black) or in the presence of 200 µM CBD (red).

To address this issue, we evaluated the possible interaction between these two antimicrobials using NMR (nuclear magnetic resonance) and we observed that these two molecules interact with each other in a stoichiometric relationship of 1:1. We observed that there is equilibrium where exchange is rapid (on the microsecond scale). In principle, at the 1:1 equivalent condition, all CBD and colistin molecules are interacting with a regime of rapid exchange (Figure 8 and Figure 9). These results provide evidence, for the first time, of a specific interaction between CBD and colistin, likely involving at least one residue. Because colistin contains multiple amide protons with similar intrinsic chemical shifts, the appearance of a new resolved signal strongly suggests the stabilization of a specific local conformation (or microenvironment) in one of the peptide segments upon ligand binding. This behavior is commonly observed in peptides with flexible backbones when a ligand induces local ordering or hydrogen-bond reorganization [29]. In addition, several pre-existing amide resonances exhibit small but systematic chemical shift perturbations across the titration, consistent with local electronic/environmental changes typical of non-covalent interactions in flexible peptides. The perturbations are localized in the amide region and do not reflect non-specific broadening or ethanol-dependent shifts.

While full residue assignments are outside the scope of the present work, the combination of (i) the emergence of a new amide resonance with a definable Kd, (ii) reproducible and localized chemical shift perturbations, and (iii) ligand-dependent, saturable behavior collectively supports the conclusion that CBD engages colistin through a specific and structurally meaningful interaction. These data align well with the physicochemical properties of both molecules and provide a solid foundation for future detailed mapping using multidimensional NMR approaches.

**Figure 10:**
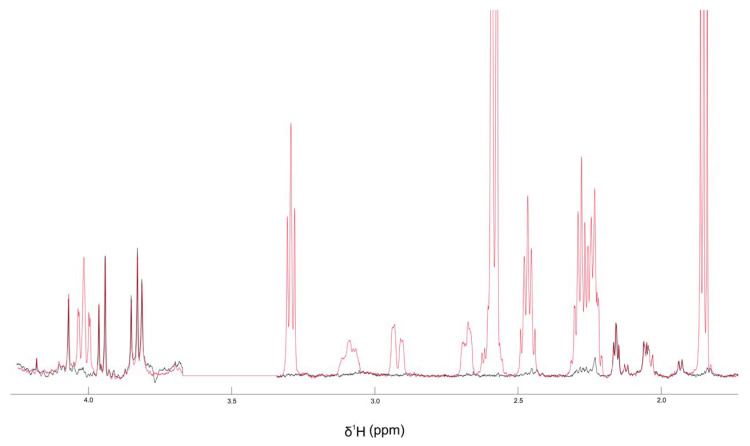
^1^H-NMR spectra of the aliphatic region (4–2 ppm) of meropenem (500 µM) in DMSO-d_6_ in the absence (black) or in the presence of 2 mM CBD (red).

It is noteworthy that this phenomenon is not observed with meropenem, in accordance with the lack of synergistic activity with this antibiotic in the microbiological assays (Figure 10).

**Figure 11:**
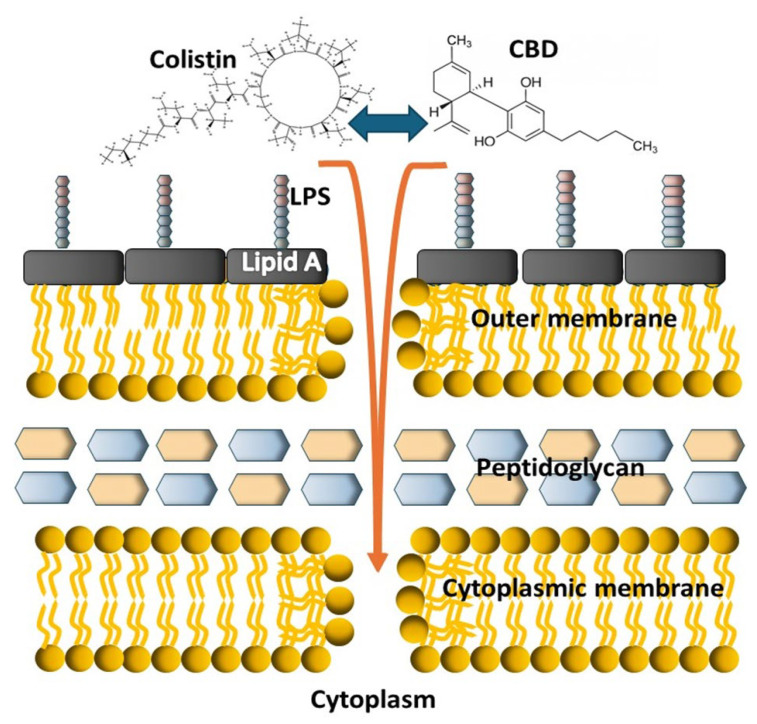
Schematic cartoon depicting the possible mode of action of the colistin–CBD combination on Gram-negative bacteria. The two molecules strongly interact with each other and then act together on the bacterial membranes at the first step of the bactericidal process.

In light of this evidence, a different approach is suggested, in which colistin and CBD do not act separately in a sequential mode of action, at least in the first steps regarding membrane interaction; on the contrary, we propose that colistin and CBD act together in a strong engagement to display synergy, probably interfering with the outer membrane of GN bacteria, initiating a bactericidal process and, as previously suggested [16], further affecting LPS, DNA, and lipid biosynthesis (Figure 11).

As the structural basis of this mechanism has not yet been fully defined, mechanism of action involving cooperative membrane insertion between CBD and colistin or, probably, a single CBD–colistin complex are possible explanations for the observed results.

## 5. Conclusions

Altogether, the results presented here unveil a crucial interaction in the very first steps of the CBD–colistin synergy mechanism, which involves a strong molecular interaction between the two molecules, highlighting that the two molecules acting together is essential for further antimicrobial activity.

## Figures and Tables

**Table 1:** The different clinical isolates with their mechanism of resistance to colistin were analyzed, comparing the MIC to colistin and CBD alone and in combination (MEAC). The synergistic combination with the minimum CBD concentration is presented.

Strain	Mechanism of Resistance	Col MIC (µg/mL)	CBD MIC (µg/mL)	MEAC	
				Col (µg/mL)	CBD (µg/mL)
*Escherichia coli* M15049	*mcr-1*	6	>256	3	1
*Escherichia coli* M15224	*mcr-1*	10	>256	2.5	2
*Escherichia coli* M27666	Chromosomal mutation	6	>256	3	4
*Acinetobacter baumannii* M27167	Chromosomal mutation	128	>256	16	0.5
*Salmonella* spp. M28629	Chromosomal mutation	4	>256	2	0.5
*Salmonella typhimurium* M22399	*mcr-1*	4	>256	2	1
*Klebsiella pneumoniae* M21664	*mcr-1*	16	>256	4	1
*Klebsiella pneumoniae* M28644	Chromosomal mutation	16	>256	4	0.5

## Data Availability

The original contributions presented in this study are included in the article. Further inquiries can be directed to the corresponding author.

## Abbreviations

The following abbreviations are used in this manuscript:

CBD	Cannabidiol.
MEAC	Minimal effective antibiotic concentration.
MIC	Minimum inhibitory concentration.
SEM	Scanning electron microscopy.
NMR	Nuclear magnetic resonance.
